# A Roadmap for Preventing Ageism in Healthcare: Perspectives from Slovenian Healthcare Professionals

**DOI:** 10.1007/s10823-026-09586-9

**Published:** 2026-06-15

**Authors:** Špela Mihevc, Katarina Galof, Danica Rotar Pavlič

**Affiliations:** 1 University of Ljubljana, Ljubljana, Slovenia; 2 Angela Boškin Faculty of Health Care, Jesenice, Slovenia; 3 University Rehabilitation Institute Republic of Slovenia, Ljubljana, Slovenia

**Keywords:** Lifelong learning, Communication, Intergenerational cooperation, Teamwork, Combating discrimination

## Abstract

This qualitative study explores Slovenian healthcare professionals’ perceptions of ageism in their work environments and their role in fostering relationships to prevent ageism with older adults. A phenomenological approach was used, grounded in hermeneutic phenomenology, and reflexive thematic analysis was used to identify key themes. Data were collected via eight focus groups from September 2024 to June 2025, involving 38 participants from various healthcare sectors. The study revealed that healthcare professionals acknowledge the prevalence of ageism and recognize their role in fostering respectful relationships with older adults. Key strategies for preventing ageism included empathy, interprofessional collaboration, and ongoing professional development. Organizational factors such as leadership and institutional culture were identified as critical in addressing ageism. Combating ageism in healthcare requires a multilayered approach, including education, organizational change, and systemic reforms. These findings highlight the need for integrated educational, organizational, and systemic interventions to support age-inclusive care in healthcare settings.

**Supplementary Information:**

The online version contains supplementary material available at 10.1007/s10823-026-09586-9.

## Introduction

Contemporary healthcare is shaped by an ageing population, rapid technological advances, shifting expectations regarding quality of life, and the evolving demands of all stakeholders, providers and users alike.

Belonging is a pervasive human need that is vital to people’s well-being as they age. Ageist attitudes and stereotyping tend to jeopardize older adults’ experiences of belonging. Experiences of belonging are strongly associated with being valued and respected (Fortune & Weisgarber, 2023). This also applies to the relationship established among healthcare professionals and older adults during medical treatment. Older adults may encounter ageist attitudes and assumptions, which cause the belief that their value diminishes with age. Seeking a sense of belonging, they are likely drawn to experiences that help them feel worthwhile and integral to their communities, living environment, hospital, or retirement home. Feeling worthwhile may come from continuous engagement in productive activities, but also from connecting with others in meaningful ways and also being appreciated for input to social exchanges within the healthcare system, and interactions with health professionals, medical students, relatives, and other informal healthcare providers (Fortune & Weisgarber, 2023).

According to the latest data, life expectancy in Slovenia is 82.7 years for women and 75.7 years for men (Statistical Office of the Republic of Slovenia [SURS], 2025). The strategy for a long-living society addresses demographic change by promoting quality of life, participation of all generations, intergenerational cooperation, and active ageing (Republic of Slovenia Institute of Macroeconomic Analysis and Development [UMAR], 2017). Population ageing brings new social challenges and changes perceptions of old age. Older adults are a heterogeneous group differing in health, interests, education, and ability to live independently and actively spend their free time.

Ageism is present in the way people think, feel, and act toward others and themselves according to age, whether they are conscious of it or not. It is everywhere – in institutions, relationships, and people themselves. Ageism harms people individually and collectively, affecting health and well-being (World Health Organization [WHO], 2021). A systematic review by Chang et al. (2020) on the global reach of ageism on older adults’ health included over 7 million participants and is the most comprehensive review of health consequences of ageism to date, demonstrating the pernicious reach of ageism. Ageism led to significantly worse health outcomes in 95.5% of studies and in 74.0% of the 1,159 associations examined between ageism and health.

A wide-ranging review of international perspectives on ageing and perspectives on ageism (Wyman et al., 2018) provides an ample body of literature documenting negative attitudes toward older adults among healthcare professionals and another aspect of age discrimination related to the way healthcare professionals communicate with older adults. They clearly state the factors associated with healthcare professionals and the factors associated with older adults. They do not neglect the structural factors that impact care for older adults regarding ageism at various levels (meso and macro).

In Slovenian qualitative research, authors have identified barriers to cultural competence and measures for its advancement pertaining to several areas, including staffing, information for healthcare users, multidisciplinary and multi-level approaches, data collection and research, communication opportunities and skills, the legislative foundation, flexibility of the healthcare system, quality standards, and educational efforts and policies (Halbwach et al., 2025). Research on ageism is limited, with only one survey identified as investigating ageism in Slovenian healthcare clinical settings and confirming the existence of ageism in clinical settings, with more than half of the respondents having experienced at least one discriminatory event (Lešnik & Tomažič, 2017).

Findings in a systematic review and meta-analysis by Burnes et al. (2019) suggest that feasible strategies involving education and intergenerational contact can serve as a basis for effective interventions to reduce ageism. Interventions must be developed and adopted for different national and cultural contexts.

If quality healthcare is to meet the diverse needs of older adults, more attention needs to be paid to understanding the views of older adults. Healthcare professionals need training to reduce ageism, emphasizing empathy and effective communication with older adults. Awareness of ageism should be integrated into healthcare curricula to shape inclusive healthcare professionals. Research should focus on gaining a better understanding of the impact of ageism and developing effective interventions, and policies must ensure equal and inclusive access to services for older adults.

Within the Slovenian context, this study is groundbreaking. It gathers expert opinions for preparing recommendations to avoid ageism in healthcare and offers avenues for further research and clinical practice.

## Methods

A qualitative research methodology with a phenomenological approach was used. This type of research aims to explore the underlying structures of the “lived experience” of a particular phenomenon and to understand how it is shared by multiple people (Creswell, 2013). This study specifically applied van Manen’s hermeneutic phenomenology (van Manen, 2016). Hermeneutic phenomenology is a research method focusing on the production of meaning. This approach was selected not only for its ability to capture the voices of healthcare professionals and illustrate their experiences, but also for its capacity to understand these experiences within the broader context of their working environment. This methodology is widely used in health and education research (Creswell, 2013).

This study was reviewed and approved by the Slovenian Medical Ethics Committee (April 13, 2023; approval no. 0120 − 74/2023/3).

### Research Objectives and Questions

The aim of the study was to investigate whether healthcare professionals perceive ageism in their work environments, how they understand their role in establishing relationships that avoid ageism with older adults in healthcare, and whether they have the knowledge and skills to prevent ageism in their work environments.

The research questions were:

- [RQ1] What are the views of healthcare professionals regarding ageism in their work environments?
- [RQ2] What role do healthcare professionals play in establishing relationships with older adults to avoid ageism in healthcare?
- [RQ3] What strategies do healthcare professionals use to prevent ageism?

### Participants and Sampling Strategy

A total of 38 healthcare professionals between the ages of 25 and 61, with an average length of service of 18 years, including 16 years of working with older adults, took part in the study. The participants that met the inclusion criteria were specifically invited to take part in the focus groups.

Inclusion criteria:

- Employment at one of the levels of healthcare.
- Written consent to participate in the study.
- Willingness to comply with the research protocol.

Exclusion criteria:

- Healthcare professionals that do not encounter older adults in practice on a daily basis.

Participants were invited to take part in focus groups based on targeted selection criteria relating to different areas of employment at various levels of healthcare (primary = community health centers and private practice, nursing homes; secondary = general hospitals and outpatient specialist clinics; tertiary = university clinical centers and specialist rehabilitation facilities), their role in the healthcare team, and their availability. Invitations to participate were sent via announcements on their professional associations’ websites, and upon expressed interest also via email.

The researchers’ professional networks also assisted with recruitment of individual healthcare professionals within their working environments by providing an invitation to cooperate in the research. When an initial low response was identified within a particular group (doctors), they were also asked to support recruitment. The participants were diverse in terms of age, education level, profession, length of service, and the level of healthcare activities they operate in. They had experience with a wide range of older adults at various levels of healthcare and with different health conditions and diagnosis. All participants signed a consent form prior to the beginning of the study, agreeing to participate voluntarily and adhere to the research protocol (Davis, 2016), and they were informed in advance that declining or withdrawing would have no consequences. The demographic data of all participants were collected during the study. Numbers were used in the analysis and presentation of the results to ensure participants’ anonymity. No supervisory personnel were present during recruitment or data collection. To prevent hierarchical dynamics within focus groups, care was taken to ensure that no participant was in a direct supervisory role over another participant within the same group.

### Data Collection

Data were collected from September 2024 to June 2025. Eight focus groups were conducted during working time and in the working environment of the 38 participants. On average, five healthcare workers participated in an individual focus group. Participants’ characteristics are shown in Table 1.

**Table 1:** Characteristics of the participants

Participant	Birth year	Years of service	Years working with older adults	Healthcare level	Education
1	1987	12	12	Primary	PT (physiotherapist)
2	1990	9	9	Primary	PT
3	1981	8	8	Primary	PT
4	1983	17	17	Primary	PT
5	2000	2	3	Primary	PT
6	1993	3	6	Primary	PT, kinesiologist
7	1980	18	20	Tertiary	PT
8	1989	8	18	Tertiary	PT
9	2000	1	6	Tertiary	PT
10	1985	16	16	Tertiary	OT (occupational therapist)
11	1988	10	14	Tertiary	PT
12	1991	9	9	Primary	PT
13	1986	14	13	Primary	OT
14	1985	13	5	Primary	Nurse
15	1976	20	20	Primary	Nurse
16	1986	10	10	Primary	Social worker
17	1993	8	8	Primary	PT, OT
18	1998	1	1	Primary	PT
19	1978	21	21	Primary	PT
20	1972	25	25	Primary	Nurse
21	1974	24	24	Primary	MD (doctor of medicine)
22	1971	28	28	Primary	Pharmacist
23	1978	25	1	Primary	Nurse
24	1991	7	7	Primary	MD
25	1964	39	38	Tertiary	PT
26	1964	38	38	Tertiary	PT
27	1985	15	13	Tertiary	PT
28	1999	2	2	Tertiary	PT
29	1984	17	19	Tertiary	PT
30	1964	40	35	Tertiary	PT
31	1980	21	7	Secondary	OT
32	1975	24	7	Secondary	OT
33	1979	21	12	Secondary	OT
34	1980	22	18	Secondary	OT
35	1977	25	25	Tertiary	Nurse
36	1971	33	33	Tertiary	Nurse
37	1964	39	20	Tertiary	Nurse
38	1978	22	22	Tertiary	Nurse

Note: *PT* physiotherapist, *OT* occupational therapist, *MD* doctor of medicine

The process was guided by key questions formulated based on a review of national and international literature (a detailed formulation of key questions is provided in Online Resource 1). The focus group guide covered 13 key topic areas: priorities and needs of older adults; equality in the healthcare system; sources of stress and professional responses; the definition and understanding of ageism, including prejudice, stereotypes, and discrimination; factors influencing the prevalence of ageism in healthcare organizations; differences in communication with older versus younger patients; personal experiences of communicating with older people; confidentiality and data protection; emotional responses to working with older people; quality assurance in team-based care; and perspectives on formal and informal education. The guide was used flexibly; the facilitator employed probing questions to explore emergent topics when these were deemed relevant to the research questions. The flexibility of a semi-structured format allowed the interviewer to pose questions not on the interview guide if they were deemed relevant to the research (Creswell, 2013).

Before participating in the study, participants received a detailed explanation of the research project and had the opportunity to ask questions. Focus groups were recorded with their consent (Braun & Clarke, 2013). Some short final conversations took place after the recorder had been turned off and, in these cases, notes were taken to document important ideas (Silverman, 2016). Focus groups were conducted by one researcher, and a second one was responsible for technical support. All focus groups were conducted face to face, in a private room within or directly adjacent to the participants’ workplace, ensuring a quiet and confidential setting. At the beginning of each focus group, the researcher established explicit ground rules: participants were asked to treat all information shared by others as confidential and not to repeat it outside the group. Sensitive details in transcripts were anonymized during transcription. Audio recordings and transcripts were stored on password-protected institutional storage accessible only to the research team. Data will be retained for 10 years following publication in accordance with institutional guidelines; audio recordings will be securely deleted following the mandatory retention period, and anonymized transcripts may be kept for archival purposes. The duration of the focus groups ranged from 34 to 80 min, resulting in a total of 643 min of recorded material. From these recordings, a nearly verbatim transcript of 131 pages was compiled by the first author. All parts of the focus groups not relevant to the research were excluded from the analysis (Braun & Clarke, 2013). Interviewing was concluded when no new themes were presented during focus groups (Ahmed, 2025; Rahimi & Khatooni, 2024).

### Data Analysis

The data collected were analyzed using the reflexive thematic analysis method developed by Braun and Clarke (2022). Reflexive thematic analysis within the qualitative paradigm requires researchers to take an active role in developing codes and themes (Braun & Clarke, 2013; Sundler et al., 2019). Reflexivity throughout the process encourages critical examination of the data from a professional, theoretical, and personal research perspective. Each focus group was analyzed separately, before proceeding to the next, to ensure a reflexive approach focusing on process and change (Braun & Clarke, 2022). The study adopts a constructivist–interpretivist epistemological position, consistent with both van Manen’s hermeneutic phenomenology and Braun and Clarke’s reflexive thematic analysis. Hermeneutic phenomenology provided the overarching orientation, attending to lived experience, meaning-making, and the contextual embeddedness of participants’ accounts. Reflexive thematic analysis provided the systematic analytical procedure for working with focus group data. This combination is increasingly used in research on the health professions because hermeneutic phenomenology does not prescribe a single analytic method, and thematic analysis can be applied in a way that honors phenomenological principles of openness and contextual interpretation (Sundler et al., 2019). Emphasizing openness, questioning pre-understanding, and adopting a reflective attitude were identified as important methodological principles that guided researchers throughout the analysis and help uphold scientific rigor and validity (Sundler et al., 2019).

The research team included healthcare professionals and educators with professional experience in gerontology and healthcare. No member of the research team had a direct supervisory or hierarchical relationship with any participant.

The transcripts were checked against audio recordings for accuracy and read multiple times for familiarity and to gain an in-depth understanding of the content, with initial notes taken. The data were then imported into Atlas.ti (Friese, 2019) for initial coding and organized into meaningful units. Codes were grouped into potential categories and subcategories, which were reviewed and refined, and a thematic map was created and tested several times (Braun & Clarke, 2013; Saldaña, 2016). During the process, parts of the focus groups were also coded by the second and third author, and the terminology of the categories was harmonized (Guest et al., 2012). Interpretive discussions were held regularly among all three researchers.

The categories were further refined, and definitions were developed. Finally, a report was produced, including excerpts from the interviews to support the findings, ensuring that the statements quoted retained context to preserve the richness of the data (Creswell, 2013). In developing the categories, preference was given to a data-driven, inductive approach, and the imposition of a preexisting theoretical framework was avoided (Saldaña, 2016).

In the second stage, a cross-cutting interpretive synthesis was performed across all four themes to specifically address the following research question: What role do healthcare professionals play in establishing relationships with the aim of preventing ageism in the treatment of older adults? This approach draws on the principles of qualitative interpretive synthesis (Thorne et al., 2004), in which findings from an initial analysis are re-examined at a higher level of abstraction to generate integrative conceptual categories that go beyond description. Individual subcategories were re-read across themes and re-linked according to shared interpretive meaning, yielding four overarching categories. This second-order analysis did not re-code the data but reinterpreted the relationships between themes already established, consistent with the epistemological position of constructivist–interpretivism adopted in this study (Braun & Clarke, 2022).

The analysis was conducted entirely in Slovenian. Participant quotations selected for publication were translated into English by the first author. Translation accuracy was verified by the second author through independent back-translation of a representative subset of excerpts. Where minor divergences were identified, final wording has been edited for professional language by a native speaker and professional translator to ensure faithful preservation of the original meaning.

## Results

The analysis proceeded in two stages. In Stage 1, the full dataset was analyzed using reflexive thematic analysis (Braun & Clarke, 2022). This stage yielded four themes with 22 subcategories (a detailed formulation is provided in Online Resource 2). Stage 1 addressed all three research questions: perceptions of ageism (RQ1), the relational role of professionals (RQ2), and strategies used (RQ3). In Stage 2, a cross-cutting interpretive synthesis was performed across all four themes to specifically address the second research question. This second-order analysis re-examined and linked individual subcategories at an interpretive level. It went beyond description to illuminate the meanings participants attributed to their experiences (Braun & Clarke, 2006). Stage 2 yielded four broader analytic units, hereafter referred to as ‘overarching categories’. The results in Table 2 are presented through the prism of these overarching categories.

**Table 2:** Thematic framework: overarching categories, subcategories, and connecting themes

Overarching Category	Subcategories	Connecting Themes
Personal foundations for working with older adults	Values and upbringing, empathy as a personal trait, mindfulness in practice, suitability for the profession, respectful attitudes toward older adults	Discrimination
Professional knowledge and lifelong learning	Formal gerontological education, communication skills training, intergenerational knowledge exchange, peer-based learning, mentorship and role modelling	Understanding older adults
Teamwork and organizational culture	Interprofessional collaboration, team cohesion, leadership role, institutional hierarchy, information sharing, leadership accountability	Teamwork
Barriers to inclusive care	Digital exclusion of older adults, time constraints, workforce shortages, performance metrics, lack of person-centered care	Healthcare system

This approach allows a cross-sectional analysis and synthesis of content already identified to shed light on a specific research question. It is based on an interpretative level of analysis, in which individual subcategories were re-examined and linked into broader units of meaning. This approach goes beyond the purely descriptive level of thematic analysis and allows a better understanding of the meanings that participants attribute to their experiences (Braun & Clarke, 2006). The results are presented below through the prism of these overarching categories, which are supported by the statements of research participants (FG = focus group, p = participant, # = number in Atlas.ti). Additional quotes supporting the development of categories are available in Online Resource 3.

### Personal Foundations for Working with Older Adults

Attitudes toward older adults were often shaped early in life through family upbringing, social norms, and values regarding elderly care. One participant said: “We brought this from home, it’s how we were raised. We learned the relationship and respect toward the elderly and a particular relationship. You definitely acquire it in your primary family” (FG7, p4, #158). Participants noted that the environment they grew up in, as well as cultural expectations of care for their ageing parents, influenced their relationships to older adults. Another participant emphasized the importance of admission criteria before deciding what to study: “I think there should be some guides at the beginning, before you even decide to study for such a profession, to see if you’re suitable” (FG6, p3, #294). One person mentioned the impossibility of changing an individual later in the process: “A person’s education starts at the beginning; you can’t cultivate or train someone in their forties” (FG3, p2, #141). Empathy was consistently identified as a critical personal trait, described as partly innate but strengthened through professional experience as another recognized: “The way we were raised and the respect for older adults we learned at home has a big influence on how we interact with older patients” (FG2, p5, #106). Despite the challenges at the workplace, another participant recognized mindfulness within herself: “Sometimes, when I go to a patient, I catch myself feeling frantic and distressed. However, by the time I get to the patient, I disconnect and find something that relaxes me because you have to consciously program yourself. The patients can also be very difficult and challenging – not everyone – but you have to be present and calm for that person in front of you, and that’s it. All the rest has to wait” (FG2, p1, #74). Patient and tolerant healthcare professionals also influence older adults with their manner of working, according to one participant: “In many cases, I notice that, if you’re respectful toward patients and approach calmly and with tolerance, you get a very positive response from them” (FG2, p1, #10).

Collectively, these accounts suggest that the personal foundations shaping behavior preventing ageism are not solely the product of professional training but are rooted in early life experience and fundamental values. This implies that structured reflection on personal values during healthcare education is as important as formal gerontological content in shaping inclusive practitioners.

### Professional Knowledge and Lifelong Learning

Although personal experience was valued, participants stressed the importance of formal education and lifelong learning. One participant was very clear in his thinking: “I think formal education is needed to have more of these gerontologic topics included. Therefore, these topics should be dealt with not only in elective courses, but also as required subjects” (FG8, p3, #556). Another misses and recommends education on communication with older adults: “I’m sure some lecture about this would be welcome, to sort of enlighten healthcare professionals a bit more, or at least offer some ideas about what words to use or other tactics to better communicate with the elderly” (FG1, p6, #236). Intergenerational contact and exchange of knowledge among coworkers were considered essential strategies for improving practice, as some highlighted: “I feel that you learn much more when younger if you have the opportunity to work in a team where your coworkers are also a bit older with some experience in dealing with older patients. Then you can learn firsthand how they approach certain difficulties with patients and also explain situations from a different point of view, which opens your mind about it” (FG6, p3, #104). One participant emphasized that working with people from different generations enriches the approach and helps people see the patient from multiple perspectives: “She connected us all, and now we’re all trying to teach new and younger coworkers to work according to her principles because she strictly put the patient before red tape. The patient came first, basically his or her life and well-being” (FG2, p1, #60).

These accounts collectively reveal that ageism prevention through education is not a single-channel process: formal curricula provide essential gerontological knowledge, but it is the informal, relational transmission of values through intergenerational mentorship and peer learning that shapes practitioners’ day-to-day orientation toward older patients. This suggests that educational strategies aimed at reducing ageism must deliberately cultivate both dimensions: structured curriculum content and the conditions for meaningful intergenerational exchange within healthcare teams.

### Teamwork and Organizational Culture

Effective care for older adults was linked to strong team collaboration: “I think there really should be a holistic approach to treating patients. If different specialists worked together, they could solve a patient’s problem much faster. We could get together and say, OK, maybe this patient really needs treatment, say, from a psychologist. I think we’d be much more effective in our work this way” (FG1, p3, #306). In the same focus group, importance was attributed to mutual understanding and influence on the relationship with older patients: “I believe the patients here feel we’re a good team and they also come here with a positive attitude, thus returning the good vibes, which makes our work easier. Surely, there are always some exceptions” (FG1, p2, #57). The participants place great importance on open information sharing, which is not necessarily effective: “They have team meetings among themselves, like doctors, but they don’t communicate with other healthcare workers” (FG1, p1, #304). The influence of organizational leadership regarding inappropriate relationship toward an older adult is described by another participant: “The department head always reacted to irregularities, but the staff among themselves not as much. Nowadays, it depends very much on the department head; some care and deal with it, but others don’t bother” (FG7, p5, #323). Institutional hierarchy and professional priorities were significant. “The way a team is led can either promote respectful care or reinforce negative stereotypes” (FG2, p3, #323).

Taken together, these accounts suggest that neither respectful nor ageist practice emerges solely from individual attitudes; it is actively constructed or dismantled through the relational dynamics of the team. Crucially, leadership functions as a regulatory mechanism: when leaders model and enforce respectful engagement, teams transmit these norms to patients; when leadership is indifferent or inconsistent, discriminatory practices persist unchecked. This underscores that ageism prevention at the organizational level is inseparable from the cultivation of inclusive team cultures and accountable leadership.

### Barriers to Inclusive Care

Participants identified discriminatory practices during service delivery in communicating and exchanging information: “When the doctor presents them with treatment options, the patients have to think about it but they don’t really have much time” (FG8, p4, #416). In the same focus group, another participant questioned the time offered to older adults and their understanding of what was explained: “Just an explanatory duty, just how much they understand. Do they really understand what follows after the surgery?” (FG8, p3, #103). Many expressed concern that digitalization risked excluding older adults from shared decision-making. One participant expressed concern regarding older patients that have nobody: “Some manage to follow because they have grandchildren that teach them some things, whereas others don’t and, when they receive those documents and referrals, they’ve got no idea what to do” (FG1, p1, #244). Digital systems can be efficient, but they sometimes leave older patients feeling excluded from their own care decisions: “But the fact is that nowadays many more things are done online and by e-mail than before. For example, some older adults that aren’t as knowledgeable in this area may frankly be discriminated against” (FG1, p3, #253). The methods used to measure healthcare performance were seen as prioritizing the quantity of services over quality and patient experience: “I’d also say that the pace of work in the last few years can’t produce quality. The quality isn’t the same when things move so fast” (FG3, p5, #74). Similarly, according to another participant: “Sometimes, when you’re under a lot of pressure and you see a coworker slacking on duty, not reaching the same standard, you may actually reach the point when you think to yourself: I’ll do the same, who cares” (FG2, p2, #142).

What unites these diverse barriers is that they are not primarily expressions of individual prejudice but structural conditions, workload pressures, digital transitions, and workforce shortages that systematically constrain the capacity for respectful, person-centered care. This indicates that healthcare professionals may hold inclusive values while simultaneously being unable to enact them due to organizational design. Reducing ageism in this context therefore requires not only attitudinal change but structural reform to create the material conditions for dignified care.

## Discussion

Our findings echo those that emphasize the importance of education and interprofessional collaboration with a focus on development and adaptation to various contexts imperative for a quality relationship between older adults and healthcare professionals. The participants were drawn from a range of healthcare professionals, and it is not known whether the attitudes described here are reflective of the wider group of health professionals. Moreover, their attitudes were likely affected by their individual health professions. Representation was lower among nurses and doctors. Objective reasons for this were overwork and time constraints, which were also reflected in the length of the focus groups. Individual healthcare professionals responded to the invitation to participate, but they did not have the support of their coworkers and, based on the inclusion criteria, we were unable to include them in the study. The participants in the study said that they recognize a lot of ageism in their environments, but that such an attitude toward older adults is probably the reason why their coworkers did not want to participate in the study. This pattern suggests that those most concerned about ageism were most motivated to participate, whereas coworkers with more dismissive attitudes may have self-selected out. Future research should use purposive sampling strategies specifically targeting nurses and physicians to capture their distinct professional perspectives on ageism.

Reducing ageism in healthcare requires strategies that operate at both the individual and systemic levels. A recent systematic review and meta-analysis by Apriceno and Levy (2023) confirms that educational interventions and positive intergenerational contact are among the most effective and feasible approaches to reducing ageist attitudes, with the combined application of both producing the strongest effects on improving perceptions of older adults (*g* ≈ 0.33) and enhancing knowledge about ageing (*g* ≈ 0.58). These findings build on the earlier work of Burnes et al. (2019), which demonstrated that ageism reduction programs have the greatest impact when tailored to the national, cultural, and organizational context they are implemented in (Nojomi et al., 2023).

A notable finding of this study is that healthcare professionals themselves recognize the heterogeneity of older adults and their varying needs, preferences, health statuses, and social circumstances, and they identify this recognition as fundamental to non-ageist practice. This is consistent with Zisberg et al.’s (2024) evolutionary concept analysis of age-friendly healthcare, which identifies person-centeredness and the avoidance of stereotyping as core components of inclusive care. What this study adds is a practitioner-level perspective: not only do healthcare professionals endorse person-centered values in principle, but they describe concrete relational practices – taking time, listening, explaining, and adjusting communication – as the everyday enactment of those values. This suggests that the gap between ageist and non-ageist care may lie less in attitudes and more in whether organizational conditions allow these relational practices to occur.

In healthcare education, targeted programs have been shown to effectively reduce ageism among medical and allied health students, particularly when they incorporate direct interaction with older adults and structured reflection. Embedding ageism awareness into both undergraduate and graduate education can shape inclusive attitudes among future healthcare professionals. However, education alone is insufficient; systemic and organizational change is also necessary. Leadership styles, institutional culture, and resource allocation have been identified as the key factors influencing whether ageism is perpetuated or mitigated within healthcare systems (Burnes et al., 2019). At the policy level, agencies such as the World Health Organization have called for multilevel strategies to combat ageism, encompassing legislative reforms, workforce development, and public awareness campaigns (WHO, 2021). In Slovenia, this study represents a fundamental step in capturing expert perspectives to guide recommendations for preventing ageism in healthcare, with the potential to inform both future research and clinical practice.

This study demonstrates that healthcare professionals perceive the prevention of ageism as grounded in both personal dispositions and professional competencies. The emphasis on upbringing, family values, and intergenerational contact during early life aligns with recent evidence that early exposure to older adults fosters empathy, reduces stereotyping, and supports more positive attitudes toward ageing (Burnes et al., 2019; Chang et al., 2020). Empathy, described by the participants as both an intrinsic trait and a skill, refined through professional experience, reflects Kitwood’s framework, in which empathy is a cornerstone of person-centered care (Kitwood, 1997). This resonates with contemporary gerontological perspectives emphasizing empathy training as a key component of age-friendly healthcare (Zisberg et al., 2024).

Participants’ emphasis on both formal and informal pathways to knowledge challenges a common assumption in ageism-reduction literature that curriculum reform alone is sufficient. Although structured gerontological content was seen as necessary, participants consistently pointed to intergenerational mentorship and peer learning as the mechanisms through which inclusive attitudes were most durably transmitted – not through formal instruction, but through working alongside experienced coworkers that modeled patient-centered values. This finding aligns with Apriceno and Levy’s (2023) evidence that intergenerational contact produces stronger effects on ageist attitudes than education alone, and it extends it by showing that such contact is not only a formal intervention strategy but an organic feature of well-functioning healthcare teams. It also reinforces the perspective of Burnes et al. (2019), who argue that effective ageism-reduction programs must be adapted to the specific relational and cultural context in which practitioners work – a condition that peer-based learning within teams naturally fulfills.

The identified importance of interprofessional teamwork and shared organizational culture corresponds to interprofessional care research showing that cohesive teams and open communication channels contribute to more respectful and inclusive care environments (Wyman et al., 2018; Reeves et al., 2017). Critically, however, the participants in this study did not simply describe teamwork as beneficial; they identified the absence of cross-professional communication as a specific mechanism through which ageism is reproduced. Doctors’ team meetings that excluded other health professionals, or department heads that failed to address discriminatory behavior, were experienced not as neutral gaps but as active enablers of ageist practice. This nuance goes beyond what is typically reported in interprofessional collaboration research and suggests that teamwork quality is not only a predictor of care quality but a structural determinant of whether ageism is challenged or normalized within a given setting.

Organizational factors emerged as both enablers and barriers to inclusive care. Leadership style, institutional culture, and hierarchical structures influence whether ageism is perpetuated or addressed within healthcare settings – findings consistent with the WHO’s *Global Report on Ageism* (2021) and corroborated by recent organizational research, highlighting the role of transformational leadership in fostering age-inclusive policies (Bradley, 2020).

Concerns about digitalization echo current debates on the “digital divide” in healthcare, in which older adults with limited digital literacy risk exclusion from decision-making and equitable service access (Wu et al., 2025). Similarly, workforce shortages and performance metrics focused on service throughput rather than care quality can indirectly reinforce ageism by constraining time for personalized interactions – an issue also noted in recent European policy reviews on healthcare workforce sustainability (OECD, 2024).

Collectively, these findings indicate that preventing ageism in healthcare is not solely a matter of changing individual attitudes but requires multilevel strategies that integrate education, supportive team cultures, inclusive leadership, and systemic design changes. This perspective aligns with calls from global health authorities for comprehensive approaches addressing both micro-level interactions and macro-level policy frameworks (Fragoso & Fonseca, 2022).

### Implications for Practice and Policy

This study highlights the need for a multi-layered approach to combat ageism in healthcare. At the educational level, structured training on ageism should be integrated into undergraduate, graduate, and ongoing professional development, focusing on empathy, intergenerational contact, and person-centered communication (Apriceno & Levy, 2023; Nojomi et al., 2023). Reflective practice and peer learning can challenge stereotypes and foster inclusivity (OECD, 2024).

At the organizational level, leadership that values respect, transparency, and inclusivity is essential. Transformational leadership and supportive institutional cultures are linked to lower discriminatory behavior and higher staff engagement (Bradley, 2020; Zisberg et al., 2024). Reviewing hierarchical structures to promote inclusivity in service delivery is also critical.

At the system level, health policies should shift their focus from throughput to care quality and patient experience, while addressing workforce shortages (OECD, 2024). Digitalization strategies should include training and support for older adults facing online barriers (WHO, 2021; OECD, 2024).

### Future Research Directions

Future research should explore healthcare professionals’ and older adults’ perspectives to better understand how ageism is experienced and addressed in care settings. Comparative studies across cultures and healthcare systems could reveal how structural factors influence ageist attitudes (Burnes et al., 2019). Intervention studies are needed to test ageism-reduction programs in Slovenian healthcare, including blended approaches combining education, intergenerational initiatives, and organizational change (Apriceno & Levy, 2023). Longitudinal studies could track how attitudes evolve over time in relation to workplace culture and policy changes (Reeves et al., 2017; Fragoso & Fonseca, 2022). Policy research should assess the impact of legislative reforms on patient outcomes and service accessibility.

### Strengths and Limitations

This study’s strength lies in its focus on ageism within Slovenian healthcare, offering diverse perspectives across health professions. The use of hermeneutic phenomenology provided in-depth insights into lived experiences, and reflexive thematic analysis ensured data-driven findings. A limitation of the study is the unequal representation of professional groups. Although individual representatives of these groups expressed interest in participating, focus groups could often not be organized due to the unwillingness of their coworkers to participate and scheduling difficulties. A distinct limitation concerns self-selection bias. Individuals with experiences of ageism or stronger opinions on the topic were likely more motivated to participate than those without such experiences or views. Due to the sample size, the findings are specific to this context, but there is potential for their transferability to similar environments or situations, provided that relevant similarities are considered. Representation of nurses and physicians was lower due to time constraints, and reliance on self-reported data may have led to social desirability bias. Focus groups, although rich in interaction, may have constrained the expression of critical views on sensitive topics such as workplace discrimination.

## Conclusion

This study sheds light on healthcare professionals’ perceptions of ageism in Slovenian healthcare settings, their roles in fostering respectful relationships with older adults, and the systemic factors that shape these interactions. The findings suggest that preventing ageism requires more than individual awareness; it demands a coordinated multilevel approach that integrates education, organizational culture change, and supportive policies.

Key strategies include embedding ageism-awareness training in healthcare curricula, fostering intergenerational contact, strengthening teamwork, and ensuring that digitalization processes remain inclusive for all patients. Leadership and management practices that promote respect, collaboration, and empathy within healthcare teams are critical in creating environments in which older adults are valued and actively included in care decisions.

Future research should connect the perspectives of healthcare professionals with the lived experiences of older adults, making possible the development of targeted context-specific recommendations to prevent ageism. Such efforts have the potential to improve not only patient satisfaction and health outcomes but also the professional fulfillment of those providing care.

## Supplementary Information

Below is the link to the electronic supplementary material.

Supplementary Material 1 (DOCX 15.9 KB)

Supplementary Material 2 (DOCX 35.0 KB)

Supplementary Material 3 (DOCX 17.9 KB)

## Data Availability

No datasets were generated or analysed during the current study.
