# Supplementary material for: A Roadmap for Preventing Ageism in Healthcare: Perspectives from Slovenian Healthcare Professionals
Source: J Cross Cult Gerontol. 2026 Jun 15;41(2):35. doi: 10.1007/s10823-026-09586-9 (PMC13265588; doi:10.1007/s10823-026-09586-9)
Supplement: Supplementary file 1 — Supplementary Material 1 (DOCX 15.9 KB) [file 10823_2026_9586_MOESM1_ESM.docx]

**Online Resource 1:** **Key questions**

1. In your opinion, what are the priorities of older adults with regard to healthcare?

2. How do you see their needs being met?

3. Please describe how equal older adults are compared to younger people in the healthcare system.

4. In what areas do you think they experience the most stress and how can you help them?

5.How would you define ageism (sub-question on prejudice, stereotypes, and discrimination)?

6. What factors influence the prevalence of ageism in healthcare organisations?

7. In your opinion, how does communication with older adults differ from communication with younger people?

8. What are your experiences of communicating with older adults?

9. How is confidentiality and the protection of personal data handled when dealing with older adults?

10. What do you experience when dealing with older adults?

11. What fulfils you and what makes you sad?

12. How does your team ensure the quality of services for older adults?

13. What do you think of formal and informal education in dealing with older adults?
