# Supplementary material for: A Roadmap for Preventing Ageism in Healthcare: Perspectives from Slovenian Healthcare Professionals
Source: J Cross Cult Gerontol. 2026 Jun 15;41(2):35. doi: 10.1007/s10823-026-09586-9 (PMC13265588; doi:10.1007/s10823-026-09586-9)
Supplement: Supplementary file 2 — Supplementary Material 2 (DOCX 35.0 KB) [file 10823_2026_9586_MOESM2_ESM.docx]

**Online Resource 2: Figure 1: The overarching categories**
