# Supplementary material for: A Roadmap for Preventing Ageism in Healthcare: Perspectives from Slovenian Healthcare Professionals
Source: J Cross Cult Gerontol. 2026 Jun 15;41(2):35. doi: 10.1007/s10823-026-09586-9 (PMC13265588; doi:10.1007/s10823-026-09586-9)
Supplement: Supplementary file 3 — Supplementary Material 3 (DOCX 17.9 KB) [file 10823_2026_9586_MOESM3_ESM.docx]

**Online Resource 3:** **Additional participant quotes illustrating the development of categories**

**Personal foundations for working with older adults**

Participant FG2, p2(#138): *“Perhaps the upbringing, from a very young age. My colleagues and I have already come here with certain values, while the now retired leader only expanded these values. You have to come here with values, you have to be raised on them.”*

Participant FG1, p6(#130): *“Whether you grew up in the presence of your grandparents that also affects you, and what experiences you have from your childhood, watching your parents’ relationship with their grandparents or your mother towards her mother in-law. That is what you learn and then mirror, or change.”*

Participant FG6, p3(#294): *“And perhaps there really should be some selection at the beginning of thew studies, some tests or oral examination to see if someone has the ability to develop compassion for fellow human beings or not, as not people are alike, so I feel that is missing in formal education.”*

Participant FG1, p2(#47): *“At the end of a therapy patients often comment: “Oh, that therapist was so kind” and you think you didn’t really go the extra mile, that there was no special success, but even just that little meant a lot to them.”*

**Professional knowledge and lifelong learning**

Participant FG4, p1(#224): *“I think we need communication, the level of communication, the type of communication, people who work with older adults need to be taught patience and the level of communication.”*

Participant FG7, p5(#186): *“We were educated and continued to educate relatives and employees how to communicate with, for example, people with dementia or age-related depression. This also includes techniques which have to be considered with other older adults.”*

Participant FG8, p4(#577): *“I believe that education adds something to your already positive or negative attitude towards old age. It is within a person; it is the energy.”*

**Teamwork and organisational culture**

Participant FG4, p5(#247): *“It often happens that your lead is amazing, but there is that one person within the team who causes trouble, and the system is already so stuck in a loop that you would need to destroy everything and rebuild it with new people.”*

Participant FG8, p1(#387): *“Critically thinking, we, healthcare professionals, are in such a hurry. It happens so many times that you clearly explain everything to the patients, and they are just looking at you, and then some time later, they ask the same question to another nurse, and you get angry because you told them everything half an hour ago.”*

Participant FG5, p1(#173): *“And don’t even have the possibility to take time, because everything is set up so that we meet the norm.”*

Participant FG1, p3(#7): *“Personally, I notice that kindness means a lot to patients, to listen to them and immediately offer advice, to explain what we will do and guide them through the treatment process.”*

Participant FG8, p2(#416): *“We do, however, call it as it is. When they come to the department, we bombard them with information, impersonal orders, such as: “ok, so surgery is tomorrow, here, sign the agreement document”, and protocols, these beautiful protocols, all in a hurry, so formal.”*

Participant FG1, p3(#93): *“It often happens that we have to repeat something many times. And perhaps they come the next time with the same question, or you see they did not comprehend, and you just explain it again. It is visible on their faces when they actually understand.”*

**Barriers to inclusive care**

Participant FG1, p1(#244): *“Some nurses offer their help and say: “Should I make an appointment for you?”, while others just hand the referral and goodbye: “do it at home”. And then the patients come with referrals and ask: “Is this one for you, which one for the next medical examination, where do I make the next appointment?””*

Participant FG6, p5(#328): *“They don’t know how it works, how to do it, they are lost. There are some associations, such as Simbioza, which work on digital literacy, but many older adults are not included.”*

Participant FG1, p3(296): *“All these apps for booking appointments and accessing doctors – and every institution has its own – mean that nurses answer the phone less often. I think it's much easier for older adults to have a direct conversation. For healthcare workers, on the other hand, this is much more challenging because older adults just want to solve their problems over the phone. In fact, technological developments have made this very complicated for older adults.”*

Participant FG2, p3(#16): *“I think it is very important, and it means a lot to older adults, that you take time, the time which is lacking for them, as they are offered less and less of it.”*

Participant FG2, p1(#50): *“For example, these are the patients who cannot get up and are restless, so they automatically start shouting. Sometimes, it is enough if you just stop and ask if the are ok or need anything. Perhaps they only need to tell you something, lay back and fall asleep. But not all healthcare professionals have or take the time to do so, less and less anyway.”*

Participant FG2, p5(#22): *“To really check if they understand, not like at the doctor’s visit when they go from one to another, but to really ask them if they have any questions, if they understand everything.”*
